# Reliability of mixed dentition space analysis using a digital model obtained from an optical impression: a preliminary study

**DOI:** 10.1186/s13104-023-06678-4

**Published:** 2024-01-03

**Authors:** Ayuko Okamoto, Hiroyuki Karibe, Satoshi Tanaka, Tomomi Kawakami, Akikazu Shinya

**Affiliations:** 1 Department of Pediatric Dentistry, School of Life Dentistry at Tokyo, The Nippon Dental University, 1-9-20 Fujimi Chiyoda-ku, Tokyo, 102-8159 Japan; 2 Department of Dental Materials Science, School of Life Dentistry at Tokyo, The Nippon Dental University, 1-9-20 Fujimi Chiyoda-ku, Tokyo, 102-8159 Japan

**Keywords:** Arch length discrepancy, Mixed dentition, Model analysis, Optical impression

## Abstract

**Objective:**

While mixed dentition space analysis is a common practice in pediatric dentistry, digital models created using an intraoral scanner are not as widely used in clinical settings. This preliminary study used a very small sample size with one reference model and aimed to (1) compare the accuracy of mixed dentition space analysis using a digital model obtained from an optical impression with that of conventional plaster model-based analysis and (2) assess inter-examiner differences.

**Results:**

The space required for the mandibular permanent canine and premolars and arch length discrepancy were calculated using each model. The largest significant difference between plaster- and digital model-based analyses was identified when the right arch length discrepancy was considered (-0.49 mm; 95% confidence interval: -0.95–0.03); however, the value was considered clinically insignificant. Significant inter-examiner differences were observed for six items of the plaster model; however, no such differences were observed when using the digital model. In conclusion, digital model space analysis may have the same level of accuracy as conventional plaster model analysis and likely results in smaller inter-examiner differences than plaster model analysis.

## Introduction

Crowding is a common issue among children with mixed dentition. Anterior crowding is identified as the discrepancy between the interdental width of the four permanent incisors and the available space of the alveolar base [1]. Particularly, mandibular anterior crowding is frequently discovered by parents or dentists during the early stages of mixed dentition. [2]. The possible causative factors for mandibular anterior crowding in the early mixed dentition are premature loss of primary teeth attributed to dental caries or trauma, and fused primary teeth, which is associated with tooth misalignment [3, 4].

Crowding can negatively impact oral health by making optimal oral hygiene maintenance challenging, resulting in periodontal disease and esthetic concerns. Dental crowding was significantly associated with a high prevalence of gingivitis in 6–12-year-old schoolchildren [5]. According to a recent systematic review, the prevalence of dental crowding increased from primary to mixed dentition, rising from 16 to 37% [6]. Therefore, the presence of crowded mandibular incisors increases the risk of periodontal disease [7]. Moreover, dental crowding has been found to have a significant impact on the quality of life and self-esteem related to oral health [8]. This finding indicates that crowding is involved in dentofacial esthetics. Nevertheless, a recent study revealed that treating crowding in children will improve the psychological health, well-being, and body image in adulthood [3].

When considering interventions for mandibular incisor crowding in the mixed dentition, it is important to take into account the anatomical limitations of the mandibular arch, potential impact on the child’s quality of life, and amount of time required for treatment. In orthodontic treatment planning, the clinician can make decisions based on the likelihood of spontaneous alignment of the mandibular incisors in children with crowding up to 4 mm [9]. Space analysis is crucial for determining whether to implement early orthodontic intervention or merely monitor occlusal development during the mixed dentition period as it helps in selecting the most appropriate approach [10]. In addition, the analysis helps clinicians plan treatments based on current space measurements and tooth size predictions [11]. Thus, mixed dentition space analysis is essential for early diagnosis and successful treatment of developing malocclusions, which can yield long-lasting benefits in the quality of life in patients.

Space analysis involves accurately comparing the space available with that required to align the teeth. The analysis is performed by taking measurements using plaster models and a caliper; therefore, it may be influenced by the examiner’s measurement skills. Previous studies have reported the reliability of digital model analysis of permanent dentition [12–14]. The studies showed that most digital model-based measurements had clinically acceptable accuracy comparable with those made using a caliper to assess plaster models. A recent systematic review also reported the high accuracy of digital and alginate impression methods [15]. However, the digital model-based measurement method may affect the reproducibility of measurements [12].

Although mixed dentition space analysis is frequently performed in clinical pediatric dentistry, digital models made using an intraoral scanner are used less frequently in clinical practice. We believe it is necessary to study the usefulness of intraoral scanners and digital models to promote their use in pediatric dentistry. However, evidence on the reliability of digital model analysis in mixed dentition periods is limited, and the usefulness of digital models for space analysis in pediatric patients remains unclear [16]. Therefore, this preliminary study aimed to compare the accuracy of space analysis using a digital model with analysis using a conventional plaster model and to assess inter-examiner differences.

## Methods

**Fig. 1:**
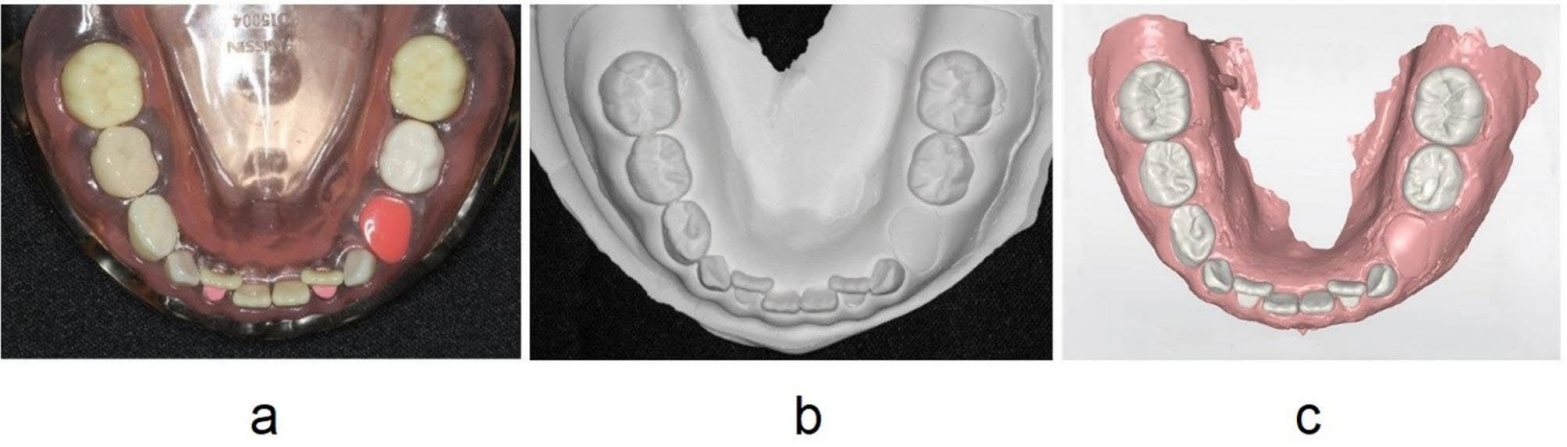
Models used in this study (**a**) A mixed dentition model for pediatric dental training used as a reference model, (**b**) a plaster model made from an impression of the reference model using alginate impression material, and (**c**) a digital model obtained from an optical impression of the reference model using an intraoral scanner are shown

A mandibular mixed dentition model for pediatric dental training (PDI5004-UL-SCP-HM, Nissin Inc., Tokyo, Japan) was used as a reference. Measurements were performed using (A) the reference model, (B) a plaster model obtained from a conventional impression of the reference model made using alginate impression material, and (C) a digital model obtained from an optical impression of the reference model made using an intraoral scanner (Primscan, Dentsply Sirona K.K., Tokyo, Japan; Fig. 1).

The mesiodistal widths of the four permanent mandibular incisors—the right lateral incisor (R2), right central incisor (R1), left central incisor (L1), and left lateral incisor (L2)—and available space for the permanent canines and premolars (AS) on both sides were measured for mixed dentition space analysis. To analyze the AS, considering the crowding of the mandibular incisors, points were marked on the dental arch by assuming the normal position of the incisors, and the distance to the mesial surface of the permanent mandibular first molar was measured. The required space for the permanent mandibular canine and premolars (RS) was calculated from the sum of the mesiodistal widths of the four incisors using the following regression formula by Ono [17] based on the Japanese population:

$$ {\text{RS}} = ({\text{R}}2 + {\text{R}}1 + {\text{L}}1 + {\text{L}}2) \times 0.548 + 8.52 + 0.56$$

The RS was subtracted from the AS to calculate both sides’ arch length discrepancy (ALD).

A licensed pediatric dentist (examiner 1) performed six reference model measurements using a digital caliper (Vernier Caliper, Matsui Seimitsu Co., Niigata, Japan), with the average of the measurements being used as the reference value. The same examiner also performed measurements on plaster and digital models. Six measurements each on plaster and digital models were performed at 1-h intervals on the same day using a caliper and dedicated CEREC Ortho SW2.0 (Dentsply Sirona K.K., Tokyo, Japan) software, respectively. A third researcher read all caliper values measured by the examiner, and the examiner performed the next measurement without verifying the previous one. Therefore, the examiner was blinded to the measurements. The accuracy of model analysis was ensured by comparing the average values of the six measurement types determined using plaster, digital, and reference models. This was a preliminary study with a very small sample size. Hence, hypothesis testing/p-values were omitted. The mean differences between the three models were calculated with 95% confidence intervals (CIs).

Next, another pediatric dentist (examiner 2) performed measurements of plaster and digital models using the aforementioned methods, allowing for inter-examiner comparisons (examiner 1 vs. examiner 2). The two examiners reviewed each measurement method of the plaster and digital model-based mixed dentition space analysis together before taking the measurement. The mean differences with 95% CIs were calculated to compare plaster and digital model measurements taken by each of the examiners. When interpreting the CI, values of zero indicate that no difference was indicated, and nonzero values indicate differences between compared items.

## Results

Table 1 presents each model’s mean (standard deviation) measurements and the mean difference (95% CI) between the two models. Comparisons of the mean plaster and digital model measurements with the reference value obtained by examiner 1 revealed significant differences for six items. Table 2 shows the mean measurements obtained by each examiner and mean inter-examiner differences for each model. Significant inter-examiner differences were observed for six items measured using the plaster model; however, no inter-examiner differences were observed for the digital model. Significant inter-examiner differences for five items were observed when plaster and digital model differences were considered.

**Table 1:** Comparison of measurements determined using three models

	Reference model	Plaster model	Digital model	Difference (plaster - reference)	Difference (digital - reference)	Difference (plaster - digital)
Measurements	Mean (SD), mm	Mean (SD), mm	Mean (SD), mm	Mean (95% CI), mm	Mean (95% CI), mm	Mean (95% CI), mm
Lateral incisor width, right	5.90 (0.00)	5.98 (0.04)	5.87 (0.10)	0.08 (0.04, 0.13)	-0.03 (-0.14, 0.08)	0.11 (0.04, 0.20)
Central incisor width, right	5.31 (0.02)	5.34 (0.04)	5.25 (0.20)	0.03 (-0.01, 0.07)	-0.06 (-0.27, 0.16)	0.09 (-0.11, 0.29)
Central incisor width, left	5.11 (0.07)	5.19 (0.02)	5.17 (0.08)	0.08 (0.01, 0.16)	0.06 (0.02, 0.10)	0.02 (-0.05, 0.10)
Lateral incisor width, left	5.87 (0.04)	5.82 (0.04)	5.78 (0.04)	-0.05 (-0.12, 0.02)	-0.09 (-0.14, -0.03)	0.04 (-0.05, 0.12)
AS, right	23.48 (0.27)	23.54 (0.20)	23.88 (0.27)	0.06 (-0.30, 0.41)	0.40 (-0.15, 0.95)	-0.34 (-0.64, -0.04)
AS, left	23.60 (0.16)	23.78 (0.08)	24.08 (0.36)	0.18 (0.01, 0.36)	0.48 (-0.02, 0.99)	-0.30 (-0.74, 0.14)
RS (calculated)	21.24 (0.03)	21.32 (0.02)	21.17 (0.17)	0.08 (0.05, 0.11)	-0.07 (-0.24, 0.11)	0.15 (-0.02, 0.31)
ALD, right	2.25 (0.27)	2.22 (0.20)	2.71 (0.42)	-0.03 (-0.37, 0.33)	0.46 (-0.25, 1.17)	-0.49 (-0.95, -0.03)
ALD, left	2.36 (0.16)	2.46 (0.08)	2.91 (0.51)	0.10 (-0.09, 0.30)	0.55 (-0.10, 1.20)	-0.45 (-1.03, 0.13)

AS: available space for canine and premolars, ALD: arch length discrepancy, CI: confidence interval, RS: required space for canine and premolars, SD: standard deviation

**Table 2:** Between-examiner comparison of mandibular measurements of plaster and digital models

	Plaster model			Digital model			Plaster -digital		
Measurements	EX 1 Mean (SD), mm	EX 2 Mean (SD), mm	Inter-examiner difference Mean (95% CI), mm	EX 1 Mean (SD), mm	EX 2 Mean (SD), mm	Inter-examiner difference Mean (95% CI), mm	EX 1 Mean (SD), mm	EX 2 Mean (SD), mm	Inter-examiner difference Mean (95% CI), mm
Lateral incisor width, right	5.98 (0.04)	5.98 (0.03)	0.00 (-0.06, 0.06)	5.87 (0.10)	5.87 (0.08)	0.00 (-0.13, 0.13)	0.12 (0.08)	0.12 (0.10)	0.00 (-0.14, 0.14)
Central incisor width, right	5.34 (0.04)	5.21 (0.04)	0.13 (0.07, 0.20)	5.25 (0.20)	5.30 (0.00)	-0.05 (-0.26, 0.16)	0.09 (0.19)	-0.09 (0.04)	0.18 (-0.04, 0.41)
Central incisor width, left	5.19 (0.02)	5.23 (0.04)	-0.04 (-0.09, 0.02)	5.17 (0.08)	5.25 (0.05)	-0.08 (-0.22, 0.06)	0.03 (0.08)	-0.02 (0.06)	0.05 (-0.09, 0.19)
Lateral incisor width, left	5.82 (0.04)	5.98 (0.03)	-0.16 (-0.20, -0.12)	5.78 (0.04)	5.75 (0.05)	0.03 (-0.02, 0.09)	0.03 (0.08)	0.23 (0.07)	-0.20 (-0.27, -0.11)
AS, right	23.54 (0.20)	24.61 (0.14)	-1.07 (-1.39, -0.74)	23.88 (0.27)	23.80 (0.13)	0.08 (-0.17, 0.34)	-0.34 (0.29)	0.81 (0.17)	-1.15 (-1.43, -0.87)
AS, left	23.78 (0.08)	24.67 (0.16)	-0.89 (-1.03, -0.74)	24.08 (0.36)	23.85 (0.10)	0.23 (-0.10, 0.56)	-0.30 (0.41)	0.82 (0.18)	-1.12 (-1.53, -0.71)
RS (calculated)	21.32 (0.02)	21.35 (0.04)	-0.03 (-0.07, 0.01)	21.17 (0.17)	21.23 (0.10)	-0.06 (-0.31, 0.20)	0.15 (0.16)	0.12 (0.13)	0.03 (-0.25, 0.29)
ALD, right	2.22 (0.20)	3.26 (0.13)	-1.04 (-1.33, -0.74)	2.71 (0.42)	2.57 (0.09)	0.14 (-0.33, 0.60)	-0.49 (0.44)	0.69 (0.08)	-1.18 (-1.63, -0.71)
ALD, left	2.46 (0.08)	3.32 (0.16)	-0.86 (-0.98, -0.72)	2.91 (0.51)	2.62 (0.10)	0.29 (-0.29, 0.87)	-0.45 (0.55)	0.69 (0.20)	-1.14 (-1.80, -0.48)

AS: available space for canine and premolars, ALD: arch length discrepancy, CI: confidence interval, EX: examiner, RS: required space for canine and premolars, SD: standard deviation

## Discussion

In this study, we compared the accuracy of the mixed dentition space analysis using a digital model obtained from an optical impression with that of the conventional plaster model-based analysis. A digital model was obtained from an optical impression made using an intraoral scanner. Similar to other three-dimensional scanners, an intraoral scanner is a device that captures direct optical impressions by projecting a light source onto the dental arch [18]. Optical impressions have several advantages, including enhanced comfort for patients, simplified clinical procedures, reduced storage requirement, real-time visualization, true color representation, and better communication with patients [15, 18]. Meanwhile, the disadvantages of optical impressions include the learning curve for adopting intraoral scanners and purchasing and managing costs.

A plaster model was obtained from a conventional impression using alginate impression material. In clinical settings, alginate impression material is placed on a tray, which is held in the patient’s mouth for several minutes till setting of the material. Once the impression has been taken, the tray is disinfected and then filled with hard plaster to create a plaster model. In this conventional impression taking procedure, certain problems may arise, including the presence of air bubbles, the rupture of the impression material, inaccurate impression tray dimensions, excessive or insufficient amount of impression material, inadequate adhesion of the impression to the tray, and impression material distortion owing to the disinfection procedure [12]. Further, the conventional impression may induce a gag reflex in some patients. However, the advantages of conventional impression taking include low cost, ease of handling, short placement time, and simple instrumentation and impression taking methods [19].

Kaihara et al. [20] compared tooth size, arch width, and length values determined in primary dentition using digital models with those determined using plaster models. The authors concluded that digital model-based analyses of primary dentition have high accuracy levels. In this mixed dentition study, comparing the plaster- and digital model-based measurements with those of the reference model revealed significant differences in six items. According to the literature, a difference in tooth size of > 0.3 mm is considered clinically relevant [21]. However, our results showed differences in six items ranging from 0.06 to 0.18 mm, indicating no clinically significant differences in accuracy. In contrast, a comparison of the measurements of the plaster model with those of the digital model revealed significant differences in the three items, with values ranging from 0.11 to 0.49 mm. Leifert et al. [22] compared the efficacy of plaster and digital models for space analysis of crowded permanent dentitions with Class I malocclusions. They concluded that the statistical difference in arch length measurement was not clinically significant (< 0.50 mm) [22]. In our study, the maximum mean difference between space analysis findings (ALD, right) of plaster and digital models was 0.49 mm. Although the method of analysis differed from that of previous reports, the values obtained in this study were similar to those of prior studies and may be regarded as clinically insignificant. Therefore, the digital model’s optical impression and analytical accuracies indicated that it was clinically useful.

Significant inter-examiner differences were identified for six items when the plaster model was measured. In this study, a model with anterior crowding was selected. Before taking the measurements, the examiners reviewed the mixed dentition space analysis measurements using the plaster and digital models. Nevertheless, the difficulty associated with measuring crowding of the mandibular incisors with a caliper and the more complex measurements required for space analysis after correcting incisor crowding may have been causes of between-examiner measurement differences. This finding suggests that while analyzing the plaster model, it is necessary to reduce inter-examiner differences via careful calibration, such as establishing measurement sites and a uniform method of caliper use.

In contrast, no significant differences were observed when the items were measured using the mixed dentition digital model with anterior crowding, wherein the measurements were performed using dedicated software. Kamimura et al. [23] investigated the inter-examiner reproducibility of digital and conventional impression techniques in permanent dentition. They observed that the digital impression technique has superior reproducibility compared with the conventional technique. Furthermore, they suggested that this advantage is independent of the examiner’s clinical experience or the patient’s oral health status, these findings are consistent with those of this study.

Significant inter-examiner differences between the plaster and digital models were observed for five items. Since the RS measurements of the two examiners did not significantly differ, inter-examiner differences were attributed to plaster model AS measurement differences. A recently published study indicated that digital measurement methods that do not rely on manual measurement with a caliper have improved accuracy [24]. Therefore, using measurement software for digital model analysis may facilitate uniform and accurate analysis, thereby minimizing inter-examiner differences. In addition, optical impressions are preferred over conventional impressions in children since they allow for better control of the gag reflex [25, 26]. Collecting digital data allows for the analysis of changes in oral condition progression without the need for storing plaster models. Hence, intraoral scanners are expected to be useful in clinical pediatric dentistry.

We believe that our preliminary study makes a significant contribution to the literature because limited studies have investigated the reliability of mixed dentition space analysis using a digital model fabricated using an intraoral scanner. In conclusion, this type of analysis using a digital mixed dentition model obtained using an optical impression may have the same level of accuracy as conventional plaster model analysis. Inter-examiner differences may be smaller in the digital model than in the plaster model-based analysis. However, more comprehensive future studies with actual pediatric patients are required to substantiate the significant results of this study. Further studies comparing the results of mixed dentition space analyses using plaster and digital models of pediatric patients are warranted to better determine their clinical usefulness.

## Limitations

This preliminary study had some limitations. First, the most serious weakness of this study was the very small sample size, with only one reference model. Therefore, it is essential to further increase the sample size to generalize this study’s results. Second, the models considered were not prepared based on the oral cavity of an actual pediatric patient. Therefore, the impact of the oral environment and patient behavior on the fabricated models and the related effects on the accuracy of the mixed dentition space analysis were not clarified. Lastly, we performed linear but not three-dimensional measurements. Three-dimensional measurement is easier on digital models than on plaster models. Therefore, future studies should consider three-dimensional assessments to more completely validate the accuracy of mixed dentition space analyses using digital models.

## Data Availability

The datasets generated and analyzed during the current study are available from the corresponding authors upon reasonable request.

## Abbreviations

AS: available space for canine and premolars
ALD: arch length discrepancy
CI: confidence interval
L1: mandibular left central incisor
L2: mandibular left lateral incisor
R1: mandibular right central incisor
R2: mandibular right lateral incisor
RS: required space for canine and premolars
